# Exposure of Wild Ruminants to *Toxoplasma gondii* in Alpine Ecosystems, NE Spain

**DOI:** 10.3390/vetsci12111101

**Published:** 2025-11-18

**Authors:** Alejandra Escudero, Maria Puig Ribas, Sonia Almería, Hojjat Gholipour, Lola Pailler-García, Natalia Sastre, Jordi Ruiz-Olmo, Santiago Palazón, Ferran Sayol, Johan Espunyes, Xavier Fernández Aguilar, Oscar Cabezón

**Affiliations:** 1 Anatomía Patológica, Departamento de Producción y Sanidad Animal, Facultad de Veterinaria, Universidad Cardenal Herrera-CEU, 46115 Alfara del Patriarca, Spain; alejandra.escudero@uchceu.es; 2 Wildlife Conservation Medicine Research Group (WildCoM), Departament de Medicina i Cirurgia Animals, Universitat Autònoma de Barcelona, 08193 Bellaterra, Spain; maria.puig@uab.cat (M.P.R.); gholipour.hojjat@uk.ac.ir (H.G.); 3 Virology and Parasitology Branch, Division of Food and Environmental Safety, Office of Applied Microbiology and Technology, Office of Laboratory Operations and Applied Sciences, Human Food Program (HFP), Food and Drug Administration (FDA), Laurel, MD 20708, USA; maria.almeria@fda.hhs.gov; 4 Department of Clinical Sciences, Faculty of Veterinary Medicine, Shahid Bahonar University of Kerman, Kerman 76179-14111, Iran; 5 IRTA, Programa de Sanitat Animal, Centre de Recerca en Sanitat Animal (CReSA), Campus de la Universitat Autònoma de Barcelona (UAB), 08193 Bellaterra, Spain; lola.pailler@irta.cat (L.P.-G.); xavier.faguilar@irta.cat (X.F.A.); 6 Unitat Mixta d’Investigació IRTA-UAB en Sanitat Animal, Centre de Recerca en Sanitat Animal (CReSA), Campus de la Universitat Autònoma de Barcelona (UAB), 08193 Bellaterra, Spain; 7 Servei Veterinari de Genètica Molecular (SVGM), Facultat de Veterinària, Universitat Autònoma de Barcelona (UAB), 08193 Bellaterra, Spain; natalia.sastre@uab.cat; 8 Departament de Territori, Habitatge i Transició Ecològica, Av. de Josep Tarradellas, 2, 6, 08029 Barcelona, Spain; ajruiol@gencat.cat (J.R.-O.); santiago.palazon@gencat.cat (S.P.); 9 Centre for Ecological Research and Forestry Applications (CREAF), 08193 Bellaterra, Spain; ferran.sayol@uvic.cat; 10 Departament de Medi Ambient i Sostenibilitat, Ministeri de Medi Ambient, Agricultura i Ramaderia, Govern d’Andorra, Edifici Les Columnes, Av. de Tarragona, 58-62, AD500 Andorra la Vella, Andorra; johan_espunyes@govern.ad

**Keywords:** *Felis silvestris*, mouflon, *Ovis aries musimon*, parasite, pyrenees, pyrenean chamois, *Rupicapra pyrenaica*, *Toxoplasma gondii*, wildcat, wildlife population health

## Abstract

*Toxoplasma gondii* is a protozoan parasite with a complex life cycle that involves warm-blooded animals as intermediate hosts and felids as definitive hosts. Its epidemiology in alpine ecosystems remains poorly understood. This study presents the first long-term investigation of *T. gondii* exposure in wild ruminants in the Pyrenees (NE Spain), where definitive hosts are scarce. Pyrenean chamois (*Rupicapra pyrenaica*; n = 1045) and mouflon (*Ovis aries musimon*; n = 115) sera collected between 2001 and 2024 were tested (Modified Agglutination Test, ELISA-IDvet, ELISA-IDEXX) for the presence of *T. gondii* antibodies. Sera from 53 chamois and 27 mouflon foetuses and hearts and brains from 38 chamois and 35 mouflon foetuses were analysed for the presence of antibodies and parasite’s DNA, respectively. Moreover, heart and brain (n = 3) and faeces (n = 91) from Pyrenean wildcats (*Felis silvestris*) were analysed for parasite’s DNA. Seroprevalence was overall low (chamois: 5.24%; mouflon: 1.74%). In multivariate analyses performed in chamois, seroprevalence variation was mainly associated with geographic origin and the diagnostic method used, with little influence of the individual traits of sex or age. No antibodies or *T. gondii* DNA were detected in chamois or mouflon foetuses. However, chamois did not show a clear age-related increase in exposure to *T. gondii*, as would be expected if indirect horizontal transmission were the predominant route. In contrast, *T. gondii* DNA was detected in brain and faecal samples from wildcats, confirming their role as definitive hosts in this ecosystem. Overall, our results reinforce the hypothesis that alpine ecosystems are environments with low *T. gondii* oocyst contamination and that the parasite most probably relies on sylvatic cycles. These results suggest that wild ruminants, such as chamois and mouflon, could serve as effective sentinels of changes in the extent of this parasite under ongoing environmental and ecological changes.

## Simple Summary

*Toxoplasma gondii* is a parasite that can infect many animals, including humans. The parasite needs felines to sexually reproduce, and spreads to many other species through oocysts’ contamination of the environment. In this study, we examined two species of wild ruminants, Pyrenean chamois and mouflon, inhabiting the southeastern Catalan Pyrenees (NE Spain). By analysing blood samples collected between 2001 and 2024, we found evidence that, although both species were exposed to the parasite over a prolonged period, *T. gondii* circulation in the Pyrenees was limited in both species. The parasite was not detected in either chamois or mouflon foetuses, yet no statistically significant age-related pattern of *T. gondii* exposure in chamois was observed. The parasite’s DNA was detected in wildcats from the same area, confirming this feline as the definitive host in this ecosystem. These results suggest that wild ruminants, such as chamois and mouflon, could serve as sentinels of the extent to which the environment is contaminated by *T. gondii*, helping us better understand how this parasite spreads in nature.

## 1. Introduction

*Toxoplasma gondii* is an intracellular protozoan with a complex life cycle that involves warm-blooded animals as intermediate hosts. Wild and domestic felids are the only known definitive hosts, able to excrete oocysts in their faeces [1]. *T. gondii* infection has been shown to cause severe disease in several wild species, with a wide range of clinical outcomes, including neurological, reproductive, digestive, and respiratory disorders [1]. *T. gondii* infection has been reported worldwide, including in isolated ecosystems where opportunities for oocyst transmission are limited due to the absence of definitive hosts [2,3,4,5]. One of such ecosystems assumed to support low rates of *T. gondii* transmission are montane areas.

Europe is home to several alpine mountain ranges. Regardless of their geographical location, these alpine ecosystems share specific regional characteristics, including a relatively cold and harsh climate, high altitudes, and a complex topography. Alpine ecosystems provide unique habitats that support rich biodiversity. Wild mountain ruminants from the Caprinae subfamily (Bovidae) are specifically adapted to alpine and subalpine ecosystems. Amongst these wild ruminants, chamois (*Rupicapra* spp.) is the most common and representative species in Europe. The Pyrenees are the only alpine biogeographical region in Spain (COMMISSION IMPLEMENTING DECISION (EU) 2022/223 of 16 February 2022). In the Iberian Peninsula, the main populations of Pyrenean chamois (*R. pyrenaica*) are distributed along the Pyrenees, with approximately 53,000 individuals shared among Spain, France, and Andorra [6]. However, their social behaviour may change depending on the time of the year, such as during the rut period, when fully mature males defend territories with groups of females [6]. In the southeastern Pyrenees, the introduced mouflon (*Ovis aries musimon*) is the main sympatric wild ruminant of chamois, occupying extensive ranges in these mountains. Chamois are gregarious mountain ruminants that typically remain above 1800 m in alpine meadows during the warmer months, performing altitudinal migrations to lower valleys in autumn [7] to exploit the tender, high-quality grass that emerges between melting snow patches. However, their social behavior behaviour varies seasonally—for instance, during the rutting period, fully mature males establish and defend territories that include groups of females [6]. In the southeastern Pyrenees, the introduced mouflon (*Ovis aries musimon*) is the principal wild ruminant species living sympatrically with chamois, occupying extensive areas across these mountains. Over the past decade, populations of European mouflon in the eastern Pyrenees have increased markedly, particularly in areas such as the Freser-Setcases National Game Reserve (NGR) in Spain, where densities have tripled (data from NGR rangers and technicians, Albert Alemany, pers. comm.).

Only two feline species, the definitive hosts of *T. gondii*, thrive in European alpine ecosystems, namely the Eurasian lynx (*Lynx lynx*) and the European wildcat (*Felis silvestris*). In the Pyrenees, only the wildcat is present, but its population density is low [8]. There, wildcat tends to select forested or shrubby habitats (1200–2000 m asl), avoiding areas where snow cover exceeds 20 cm depth [9]. No anthropic areas and urban settlements exist in these alpine areas; therefore, domestic cats are uncommon. Due to the low densities of wild or domestic definitive hosts, environmental transmission of *T. gondii* oocysts seems unlikely in alpine ecosystems.

Nevertheless, exposure to *T. gondii* has been reported in wild ruminants from European alpine ecosystems, and the horizontal transmission (ingestion of oocysts) has been suggested as the primary route of infection in these species [10,11,12,13,14,15,16,17,18]. The overall low prevalences documented in European alpine ruminants seem to support that the environment of alpine areas is less contaminated with oocysts than environments near human settlements [13,17]. However, in this epidemiological context, the role of vertical transmission in the maintenance of *T. gondii* in alpine wild ruminants remains to be evaluated. Moreover, most studies on the epidemiology of the parasite in wild ruminants in the alpine ecosystem have been based on cross-sectional designs, and the long-term dynamics of *T. gondii* infection have not been thoroughly studied. Furthermore, alpine ecosystems have been identified as highly susceptible to climate change. The effects of climate change on pathogen dynamics and their impacts on wildlife health are difficult to predict, highlighting the urgent need for continuous monitoring. Thus, retrospective long-term studies of pathogens in these ecosystems are highly valuable, establishing essential baselines for future research.

The present study aimed to evaluate the long-term dynamics of *T. gondii* exposure in sympatric alpine wild ruminants (Pyrenean chamois and mouflon) from the southeastern Pyrenees (NE Spain), to analyse the presence of the parasite on foetuses to assess the vertical transmission of the parasite in both species, and to define the role of wildcats from the Pyrenees in the epidemiology of *T. gondii*.

## 2. Materials and Methods

### 2.1. Study Area

**Figure 1:**
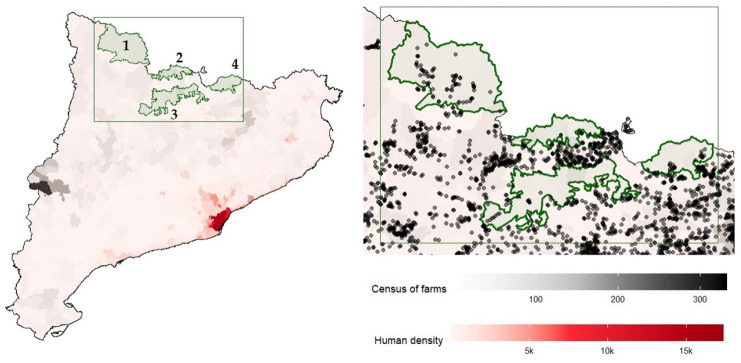
(**Left**): Map of Catalonia (NE-Spain). Study areas (1–4). Nation Game Reserves (NGR) in green: (1) NGR Alt Pallars-Aran, (2) NGR Cerdanya-Alt Urgell, (3) NGR Cadí-Moixeró, (4) NGR Freser-Setcases. Livestock farm density is represented by increasing black shading. Human population density is represented by increasing red shading. (**Right**): Black dots: livestock farms within the study area. A lower density or absence of livestock farms can be observed within the NGR compared to the surrounding areas. Data on the density of farms (cattle, sheep, goats and pigs) was provided by the *Departament d’Acció Climàtica, Alimentació i Agenda Rural* (i.e., Department of Climate Action, Food and Rural Agenda) of the Government of Catalonia.

The study area comprises the distribution of the Pyrenean chamois in the southeastern Pyrenees (Catalonia, NE Spain). Within this range, four different alpine geographical regions (National Game Reserves) were sampled: NGR Freser-Setcases, NGR Cadí-Moixeró, NGR Cerdanya-Alt Urgell, and NGR Alt Pallars-Aran (Figure 1). Several wild mountain ruminant species coexist in these areas, with the chamois being the most abundant in all cases. Other species that are also present include mouflon, red deer (*Cervus elaphus*), roe deer (*Capreolus capreolus*), fallow deer (*Dama dama*) and Iberian ibex (*Capra pyrenaica*). These mountain ungulates are managed through established hunting plans in those areas, which provide the opportunity to collect biological samples and detailed individual data.

The European wildcat is the only sympatric wild feline species of alpine ruminants in these NGRs, where its abundance and density are low [8]. These NGRs are also characterised by low human population density [19] and a sparse distribution of livestock farms (Figure 1). Both factors, human and farm densities, have been associated with a high abundance of domestic/feral cats [20] and high environmental contamination with *T. gondii* oocysts [21]. Therefore, domestic/feral cats are uncommon or absent from the study areas.

### 2.2. Animal Samples

Sera from hunted Pyrenean chamois (n = 1045) and mouflon (n = 115) were obtained between the 2001 and 2024 hunting seasons (Table 1). Information on the chamois and mouflon individuals, sex, and age based on the annual horn segments [22,23] was also recorded. Age in chamois was also classified into six categories: foetus (unborn), kids (<1 year), yearlings (1 to <2 years), subadults (2–4 years), adults prime (5–10 years), and adults old (≥11 years). In mouflon, age was classified using similar categories; however, most age records were from males, and only a single adult category was applied because precise age estimation in adults based on horn growth was less reliable.

Sera from chamois (n = 53) and mouflon (n = 27) foetuses from hunted females were also sampled. The hair distribution, the Crown-Rump Length (CRL), and the weight of the foetuses indicated that all of them were in the last third of gestation [24]. Blood was obtained by intracardiac puncture or puncture of the ophthalmic venous sinus, then centrifuged to separate the serum, which was stored at −20 °C until analysis. In addition, hearts and brains from chamois (n = 38) and mouflon (n = 35) foetuses were analysed for the presence of *T. gondii* DNA.

Also, heart and brain from Pyrenean wildcats (n = 3) were analysed for the presence of *T. gondii* DNA. These animals were found dead in the NGR Freser-Setcases, NGR Cadí-Moixeró, and NGR Alt Pallars-Aran (Catalonian Government communication). The three animals were necropsied in the Wildlife Rehabilitation Centre of Vallcalent (Lleida, NE-Spain). In addition, faeces (n = 91) from wildcats found in the Catalan Pyrenees between 2018 and 2024, and stored at −20 °C, were analysed for the presence of *T. gondii* DNA. These faeces were provided by the “*Projecte Gat Fer*” (Wildcat Project) as part of the Catalan Mesocarnivore Monitoring Scheme.

### 2.3. Serological Test

Sera from adults and foetuses were tested for the presence of antibodies against *T. gondii*. Three serological tests were used depending on the sampling period. Animals hunted between 2001 and 2008 were analysed using the commercial test Chekit Toxotest Antibody ELISA (Sensitivity 90.5%; Specificity 97.8%) (IDEXX, Westbrook, Maine, US). The results were measured as optical density percentages (OD% = (OD_sample_ − OD_negative control_)/(OD_positive control_ − OD_negative control_) × 100); positive: OD% greater than 100%, weak positive: OD% 30–100%, ambiguous: OD% 20–30%, negative: OD% < 20%. Animals hunted between 2010 and 2016 were analysed using the modified agglutination test (MAT; Sensitivity 92.6%; Specificity 95.5%) at 1:25, 1:50, 1:100 and 1:500 dilutions to detect IgG antibodies against *T. gondii* [25]. Titres of 1:25 or higher were considered positive, and doubtful results were re-examined. To eliminate particulate matter (erythrocytes, bacteria), samples were filtrated using a sterile 0.2 mm microfilter Nalgene). Previously, IgM antibodies from sera were neutralised using 2-mercaptoethanol. A commercial positive control serum (Toxotrol A, Biomerieux, Marcy-l’Étoile, France) diluted from 1:25 to 1:3200 (with a minimum titer of 1:200 in each test) and serum dilution buffer without serum as negative control were included in each test. Animals hunted between 2017 and 2024 were analysed using the ID Screen Toxoplasmosis Indirect Multi-species ELISA kit (IDvet, Grabels, France) (Sensitivity 100%; Specificity 97.56%). The results were measured as optical density percentages (S/P% = (OD_sample_ − OD_negative control_)/(OD_positive control_ − OD_negative control_) × 100); positive: S/P% ≥ 50%, doubtful: S/P% 40–50%, negative: S/P% ≤ 40%.

### 2.4. Molecular Analysis

DNA was extracted from brain (0.2 g) and heart (0.2 g) tissues of chamois and mouflon foetuses, and from the brain and heart of wildcats using the commercial kit DNeasy Blood & Tissue Kit (QIAGEN, Hilden, Germany). DNA from faecal samples (1.0 g) of wildcats was extracted using the commercial kit NucleoSpin^®^ DNA Stool Kit (Macherey-Nagel, Düren, Germany). The final elution step was performed with 50 µL of elution buffer and supernatant was diluted 1:10 for qPCR amplification. Extracted DNA was amplified using a real-time PCR (qPCR) with Toxo-SE (5′ AGGCGAGGGTGAGGATGA 3′) and Toxo-AS (5′ TCGTCTCGTCTGGATCGCAT 3′) primers, and the probe (5′ 6FAM-CGACGAGAGTCGGAGAGGGAGAAGATGT-BHQ1 3′), using a commercial kit (TaqMan^TM^ PCR Master Mix; Applied Biosystems, Carlsbad, CA, USA) [26]. Primers Toxo-SE and Toxo-SA target the 529 bp repeat region (REP529, GenBank accession no. AF146527) of *T. gondii*. The qPCR method used can detect *T. gondii* DNA extracted from a single cyst [26,27]. DNA was extracted from *T. gondii* oocysts (purchased at *Grupo SALUVET, Departamento de Sanidad Animal, Facultad de Veterinaria, Universidad Complutense de Madrid*, Spain) and used as a DNA positive control. Each qPCR run included a negative control, containing 500 µL of PBS. The cycling protocol was as follows: 50 °C for 2 min (activation of the uracil-N-glycosylase) and denaturation at 95 °C for 10 min, followed by 40 cycles at 95 °C for 15 s and 61 °C for 1 min. Samples with Ct ≤ 38 were considered positive.

### 2.5. Statistical Analyses

Differences in overall *T. gondii* seroprevalence between mouflon and chamois were assessed using Fisher’s exact test, excluding fetuses from both species. Further analyses were not performed for mouflon data owing to the small number of seropositive individuals and the presence of multiple potential confounders.

For chamois, data were explored with contingency tables and plots to assess whether there were trends, relationships, or significant differences in seroprevalence with univariate tests with age, sex, area (different NGR), year, and the method used to detect *T. gondii* antibodies. Because there were marked differences between diagnostic methods and complete separation in one of them (no positives in chamois), multivariate analyses were performed by fitting generalized linear models (GLMs) with binomial error distribution and bias-reduction estimation using the *brglmFit* method [28]. The full model included the serological status as the response variable and area, age, sex, and method as explanatory variables. For these analyses, age was categorized into 2-year intervals. Model selection was performed using an information-theoretic approach, where all possible models were compared and ranked according to the corrected Akaike’s Information Criterion (AICc) [29]. Because the method was considered a necessary control variable, it was retained in all models. Models within two AICc units of the top-ranked model were retained as the candidate set, and model averaging was performed across this set to obtain averaged parameter estimates, standard errors, and relative importance values for each predictor. Because the dataset is unbalanced regarding years and methods used to detect *T. gondii* antibodies, a similar information-theoretic approach for model selection was also done with a subset of data considering only results from MAT, the method with the highest number of observations.

Sample seroprevalence was estimated using the Wilson score method with 95% confidence intervals. All statistical analyses were conducted in R version 4.5.1 [30], using the packages *dplyr*, *epiR*, *brglm2* and *MuMIn* [31,32,33].

## 3. Results

**Figure 2:**
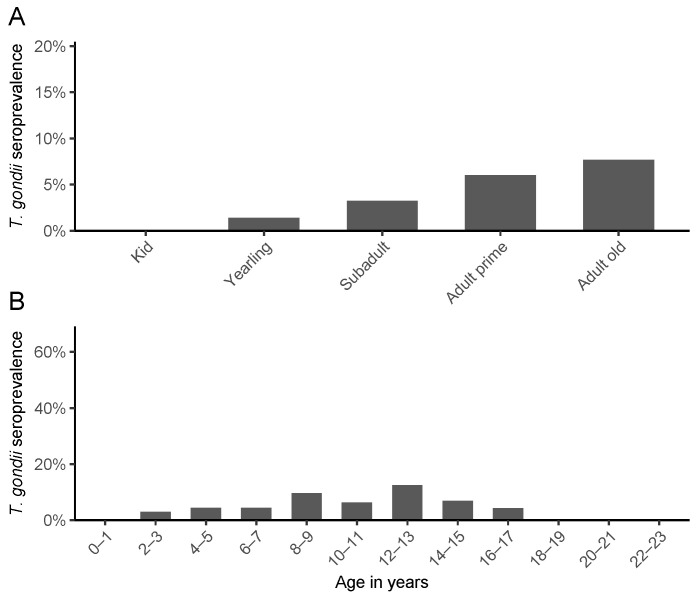
Age-related patterns of *Toxoplasma gondii* seroprevalence in chamois from the southeastern Pyrenees, Spain. (**A**) Seroprevalence by age categories. (**B**) Seroprevalence by two-year age intervals. Error bars represent 95% confidence intervals.

Antibodies against *T. gondii* were detected in 62 out of 1045 Pyrenean chamois (5.93%; 95%CI: 4.66–7.53%) and in 2 out of 115 mouflons (1.74%; 95%CI: 0.48–6.12%). Antibodies were not detected in any of the chamois or mouflon foetuses. No statistically significant differences in *T. gondii* seroprevalence were detected between species. In mouflon, sample seroprevalence was distributed heterogenously among age groups (kids 0% [n = 4], yearlings 0% [n = 16], subadults 16.67% [n = 12], adults 0% [n = 68]), and sex (females 1.23% [n = 81], males: 3.03% [n = 33]). The two subadult mouflons found positive were from the same geographic area. In chamois, significant differences in sample seroprevalence were detected with univariate tests among areas (χ^2^(df = 3, n = 1045) = 15.36, *p* < 0.01) and the method used to detect *T. gondii* antibodies (Fisher’s exact test, *p* < 0.01). No differences were detected between age groups (chamois: kids 0% [n = 17], yearlings 1.39% [n = 72], subadults 3.26% [n = 184], adults prime 6.01% [n = 549], adults old 7.69% [n = 169]), or sex (females 5.30% [n = 566], males 5.78% [n = 450]). Patterns of *T. gondii* exposure by age in chamois are shown in Figure 2. Seroprevalence data by year, area, and species are available in Table 1.

The model selection procedure identified three competing models within ΔAICc < 2, and model averaging was performed across this candidate set (Supplementary Table S1). Relative variable importance indicated that sampling area was strongly associated with serological status (importance = 0.93), whereas age and sex showed weak support (importance = 0.37 and 0.34, respectively). In the averaged model, animals from the RNC Freser–Setcases had a significantly lower probability of being seropositive compared to the reference sampling area (estimate = –1.20, adjusted SE = 0.43, *p* = 0.006). The method used to detect antibodies against *T. gondii* also had a significant effect, with samples analysed by one of the ELISAs showing a lower probability of testing positive compared to the MAT reference method in the model (estimate = –2.95, adjusted SE = 1.45, *p* = 0.041). No significant effects were detected for the remaining sampling areas, sex, or age (Supplementary Table S2). The subanalysis using only data from MAT yielded similar results on variable significance and importance.

Antibodies against *T. gondii* and parasite’s DNA were not detected in any of the chamois and mouflon foetuses analysed. However, one brain and four faecal samples from Pyrenean wildcats showed positivity for the presence of *T. gondii* DNA, representing a prevalence of 5.32% (95%CI: 2.29–11.85, 5 out of 94 wildcats analysed). Among the positive faecal samples, one sample had a very low Ct value (19.6), indicating a high DNA load, which suggests a heavy oocyst shedding by that wildcat.

## 4. Discussion

*Toxoplasma gondii* has been extensively studied in European wildlife. However, in certain ecosystems, the epidemiology of this parasite in wildlife populations is still poorly understood from a long-term perspective (i.e., over decades). The present study represents the first long-term analysis of *T. gondii* presence in alpine ruminants in the southeastern Pyrenees. Our results reveal a low circulation of the parasite in both the Pyrenean chamois and the mouflon, with little fluctuations, over the 24-year monitoring period. These results agree with previous studies on wildlife from alpine areas in Europe (Table 2). The absence of epidemic outbreaks or sudden increases in exposure aligns with previous studies suggesting that alpine ecosystems may act as protective factors against *T. gondii* seropositivity in ruminants [34].

The low prevalence observed may be attributed to the limited presence of oocysts in the environment, due to the low density of feline species in the study area. The ecology of the European wildcat, the only wild felid present in the Pyrenees, indicates avoidance of purely alpine habitats (i.e., treeless areas with frequent snow and ice cover). Additionally, the absence of domestic or feral cats, associated with low human population density (Figure 1), along with the rugged terrain and harsh climatic conditions [8], is also likely to hinder the environmental persistence of oocysts in these ecosystems.

Statistical analyses in chamois indicate that geographic origin and diagnostic method were the main factors associated with variation in seroprevalence, while individual traits such as sex and age showed little evidence of influence. Among predictors, the serological method used explained the greatest variation, with the MAT suggesting superior sensitivity and specificity, as previously reported in comparison latex agglutination test (LAT), indirect fluorescent antibody test (IFAT), or ELISA [1,25]. These findings should, however, be interpreted with caution, because different methods were applied across the study period, leading to confounding between diagnostic method and sampling periods. As a result, temporal trends in exposure to *T. gondii* or diagnostic performance of the different methods could not be reliably evaluated. Nonetheless, despite this statistical significance, the observed seroprevalence consistently reflects a low level of exposure to the parasite among these ungulates along the study period.

While a trend of increasing seroprevalence with age was observed (Figure 2), this relationship was not statistically significant in the multivariate model, likely due to low statistical power (importance = 0.37, coefficient close to 0). In a wide variety of species, *T. gondii* seroprevalence increases with age [36], supporting that indirect horizontal transmission is an important component in the parasite life cycle. In our study, however, this expected pattern may not have been detected because of the small sample size for younger and older age classes and the overall low frequency of seropositive individuals in chamois, which reduced the statistical power to demonstrate an age-related effect. The two positive mouflons were juveniles from the same geographic area, suggesting a localized exposure event. In the absence of a confirmed age-related trend or vertical transmission, the predominant transmission route of *T. gondii* in Pyrenean chamois remains unclear.

Since the first description of *Toxoplasma gondii* in 1908, numerous studies have investigated the epidemiological role of feline species worldwide [1,37,38]. However, the relative contribution of domestic and wild felids to environmental contamination with *T. gondii* oocysts depends on population characteristics, shedding prevalence, and the number of oocysts excreted. These parameters are further influenced by factors such as human activity and habitat features [39]. Therefore, fine-scale studies are essential to elucidate the role of wild felids in the environmental dissemination of *T. gondii* across different ecosystems, including the Pyrenees. The present study is the first conducted to investigate the presence of *T. gondii* in European wildcats from the Pyrenees and one of the few conducted in wildcats in Spain (Table 3) [40,41,42].

Our results, 33.3% in tissue and 4.4% in faecal samples, are consistent with the findings of Matas-Mendez et al. [40], who reported *T. gondii* in 5% of faecal samples and 50% of tissue samples from wildcats in Catalonia (NE Spain), supporting the role of the wildcat as a definitive host in this region. Oocysts of *T. gondii* can be difficult to differentiate from those of *Hammondia hammondi* or *Besnoitia* spp., and molecular analysis is needed for their detection [41]. Furthermore, the detection of *T. gondii* DNA in the faeces of the wildcats from the present study is comparable to the DNA/oocyst detection rates reported in faeces from other felids in southern Spain: Iberian lynx (0.0%; [40]), stray cats (0.0%; [43,44], and feral cats (0.7–17.0%; [45,46]). In addition, our results concur with a recent meta-analysis on the presence of *T. gondii* in domestic and wild felids which estimated the global prevalence of oocysts in faeces of domestic cats to be 2.6% (95% CI: 1.9–3.3%) and 1.21% in Europe (95% CI: 0.8–1.6%) [47]. The same study estimated the global prevalence of *T. gondii* in the faeces of wild felids at 2.4% (95% CI: 1.1–4.2%).

The detection of *T. gondii* DNA in wildcat samples further confirms their role as definitive hosts in montane areas where domestic cats are absent. While qPCR does not quantify the level of exposure, the presence of parasite DNA, whether originating from oocysts or from bradyzoites in ingested prey, indicates that the wildcat is, or is about to become, capable of shedding oocysts into the environment. Supporting this, one faecal sample exhibited a very low Ct value (19.6), suggesting a high DNA load and substantial oocyst shedding by the individual.

Although specific data on the potential reactivation of oocyst shedding in wildcats are lacking, recent studies have demonstrated that both domestic and wild felids can resume shedding under certain conditions, such as immunosuppression, coinfections, or exposure to different *T. gondii* strains [37,38]. Therefore, the role of wildcats in the environmental dissemination of *T. gondii* should not be underestimated.

Although it was confirmed that the wildcat contributes to the dissemination of oocysts and the maintenance of the parasite in the Pyrenees of Catalonia, its low population density, the fact that there is a lack of use of the alpine environments by this felid, and the limited infection prevalence support a restricted circulation of *T. gondii* in this ecosystem. Combined with the observed seroprevalence in ungulates, these findings support the hypothesis that infection in alpine species such as the Pyrenean chamois is more likely due to environmental exposure to oocysts rather than transplacental transmission.

Finally, while *T. gondii* does not currently pose a population-level health threat to any wild species in Spain, it can cause disease at the individual level under certain conditions, such as immunosuppression, infection with a virulent strain, pregnancy, or coinfection with other pathogens. Marco et al. [35] reported clinical toxoplasmosis as the cause of death in a Pyrenean chamois. In line with our findings, a molecular characterization of the *T. gondii* genotypes circulating in Pyrenean wildcats is warranted, as some authors have hypothesized that wildlife may act as a reservoir for “atypical” parasite strains [1], which could exhibit greater pathogenic potential than more common genotypes.

**Table 3:** Prevalence of *Toxoplasma gondii* infection in felines from Spain. Prevalence of infection (number of samples analyzed).

Species	Prevalence	Test	Reference
Wildcat	DNA faeces 2.64% (189) DNA tissues 33.33% (3)	qPCR qPCR	Present study
Domestic cat	14.3% (7)	serology	[48]
Feral cat (urban)	IgG 31,8% (88)	IFAT	[49]
Iberian lynx Iberian lynx Iberian lynx Wildcat Wildcat Wildcat	IgG 44.9% (69) DNA faeces 0.0% (69) DNA tissues 50.0% (60) IgG 85.0% (20) DNA faeces 5.0% (20) DNA tissues 50.0% (20)	IFAT PCR PCR IFAT PCR PCR	[40]
Feral cat (urban)	IgG 13.78% (254)	IFAT	[50]
Feral cat	Oocysts 0.7% (290)	coprology	[45]
Feral cat (urban)	IgG 42.0% (291)	MAT	[51]
Feral cat (urban)	IgG 12.28% (114)	IFAT	[52]
Wildcat	IgG 55.5% (9)	NS	[42]
Feral cat (urban)	IgG 24.2% (263) Oocysts 0.0% (263)	DAT coprology	[44]
Feral cat (urban)	IgG 53.5% (346) Oocysts 0.0% (287)	IFAT coprology	[43]
Iberian lynx	IgG 62.8% (129)	MAT	[53]
Feral cat	IgG 84.7% (59)	MAT	[54]
Iberian lynx Feral cat Feral cat	IgG 80.7% (26) IgG > 50% (25) Oocysts 17,0%	MAT MAT FA	[46]
Iberian lynx	IgG 44.0% (48)	IHA test/LA	[55]
Wildcat Iberian lynx	IgG 50.0% (3) IgG 81.5% (27)	MAT	[41]
Feral cat (urban) Feral cat Domestic cat	IgG 36.9% (317) IgG 33.3% (48) IgG 25.5% (220)	IFAT	[56]
Domestic cat	IgG 45.0% (220)	MAT	[57]
Feral cat (urban)	IgG 25.5%	IFAT	[58]

IHA: indirect hemagglutination; LA: latex agglutination; FA: direct immunofluorescence; MAT: Modified Agglutination Test; DAT: Direct Agglutination Test; NS: not specified.

From a public health perspective, both the chamois and the mouflon are game species whose meat may be consumed by humans. Although the proportion of seropositive animals is low, *T. gondii* is a zoonotic agent, and there is a potential risk of infection during carcass processing and consumption of undercooked meat. Exposure during skinning or handling of carcasses could represent a transmission risk, particularly in the absence of appropriate hygienic practices [59].

Overall, this study helps to bridge the knowledge gap regarding *T. gondii* epidemiology in montane/alpine areas, highlighting the marginal role these ecosystems may play in the circulation of the parasite in Europe. This study also confirms that horizontal transmission is the primary route of *T. gondii* infection in wild mountain ungulates, while vertical transmission likely occurs at a very low rate in these species. From an epidemiological perspective, neither chamois nor mouflon plays a significant role in completing the *T. gondii* life cycle in the Pyrenees. This is attributable to two main factors: (i) the low prevalence of infection observed, and (ii) the fact that chamois or mouflon are not a prey item for the only wild felid—and thus the definitive host—present in this high-altitude ecosystem. Further, we can conclude that the European wildcat is the main definitive host of *T. gondii* in the Pyrenees.

## Figures and Tables

**Table 1:** Prevalence of *Toxoplasma gondii* antibodies in Pyrenean chamois (*Rupicapra pyrenaica*) and mouflon (*Ovis aries musimon*) from southeastern Pyrenees (NE Spain).

Species/Year	NGR APA	NGR CM	NGR Ce-AU	NGR Fre-Set	Total	Test
***Rupicapra pyrenaica***	**12.09% (91)**	**7.90% (217)**	**12.24% (49)**	**4.12% (688)**	**5.93% (1045)**	
2001	0.00% (4)				0.00% (4)	ELISA a
2002	0.00% (8)				0.00% (8)	ELISA a
2005	0.00% (3)	0.00% (9)	0.00% (2)		0.00% (14)	ELISA a
2006	0.00% (1)	0.00% (25)			0.00% (26)	ELISA a
2007			0.00% (1)	0.00% (53)	0.00% (54)	ELISA a
2008	0.00% (1)			0.00% (87)	0.00% (88)	ELISA a
2010	0.00% (1)			8.33% (36)	8.11% (37)	MAT
2011	0.00% (1)	0.00% (34)	16.67% (6)	6.59% (91)	5.30% (132)	MAT
2012		14.81% (27)	0.00% (1)	1.78% (56)	7.14% (84)	MAT
2013	17.39% (23)	15.00% (40)	20.00% (5)	3.33% (30)	12.24% (98)	MAT
2014	0.00% (6)	0.00% (7)		5.77% (52)	4.61% (65)	MAT
2015	11.11% (9)	12.50% (32)	13.33% (15)	14.28% (28)	13.09% (84)	MAT
2016	20.00% (15)	3.85% (26)	5.88% (17)	6.60% (106)	7.31% (164)	MAT
2017	16.67% (12)	13.33% (15)			14.81% (27)	ELISA b
2018				0.00% (14)	0.00% (14)	ELISA b
2019			0.00% (2)	0.00% (22)	0.00% (24)	ELISA b
2020		0.00% (2)		4.54% (44)	4.35% (46)	ELISA b
2021				7.69% (13)	7.69% (13)	ELISA b
2022				0.00% (20)	0.00% (20)	ELISA b
2023	0.00% (1)			0.00% (22)	0.00% (23)	ELISA b
2024	16.67% (6)			0.00% (14)	5.00% (20)	ELISA b
***Ovis aries musimon***	**0.00% (11)**			**1.92% (104)**	**1.74% (115)**	
2008				6.45% (31)	6.45% (31)	ELISA a
2020				0.00% (30)	0.00% (30)	ELISA b
2021				0.00% (1)	0.00% (1)	ELISA b
2022				0.00% (9)	0.00% (9)	ELISA b
2023	0.00% (10)			0.00% (32)	0.00% (42)	ELISA b
2024	0.00% (1)			0.00% (1)	0.00% (2)	ELISA b

NGR APA: National Game Reserve Alt Pallars-Aran; NGR CM: National Game Reserve Cadí-Moixeró; NGR Ce-AU: National Game Reserve Cerdanya-Alt Urgell; NGR Fre-Set: National Game Reserve Freser-Setcases; ELISA a: IDEXX Chekit Toxotest Antibody ELISA; ELISA b: IDvet ID Screen Toxoplasmosis Indirect Multi-species; MAT: modified agglutination test.

**Table 2:** Prevalence of *Toxoplasma gondii* infection in wild ruminants from European alpine areas.

Species	% *T. gondii*	Technique	Tissue	Alpine Area/Country	Sampling Period	Reference
Pyrenean chamois	clinical case	HE/IHC	CNS, lung, liver	Pyrenees/Spain	2002	[35]
Pyrenean chamois	5.6% (n = 89)	MAT	Serum	Pyrenees/Spain	ND (1999–2020)	[10]
Pyrenean chamois Mouflon	5.99% (n = 1035) 1.74% (n = 115)	ELISA/MAT ELISA	Serum	Pyrenees/Spain	2001–2024	Present study
Alpine chamois	16.8% (n = 101) 9.27% (n = 97)	MAT ELISA	Serum	Pyrenees/France	ND	[11]
Red deer	39.5% (n = 81)	ELISA	Serum	Alps/Italy	2012	[12]
Alpine chamois	3.2% (n = 93) 2.0% (n = 50)	ELISA PCR	Serum CNS	Alps/Italy	2011–2013	[13]
Alpine chamois Roe deer Red deer Mouflon	4.0% (n = 100) 18.2% (n = 55) 18.0% (n = 50) 20.0% (n = 15)	ELISA	Serum	Alps/Italy	2017–2018	[14]
Alpine ibex	1.8% (n = 562)	ELISA	Serum	Alps/Switzerland	2006–2008	[15]
Alpine chamois Roe deer	5.0% (n = 108) 13% (n = 207)	LAT	Serum	Alps/Italy	1998–2001	[16]
Alpine chamois Red deer Roe deer	0.0% (n = 22) 0.0% (n = 13) 2.48% (n = 121)	PCR	Skel. Ms, CNS	Alps/Italy	2009–2012	[17]
Alpine chamois Mouflon Alpine Ibex	20.7% (n = 53) 30.0% (n = 10) 0.0% (n = 2)	ELISA	Serum	Alps/Slovenia	2017–2018	[18]

MAT: modified agglutination test, LAT: Latex Agglutination Test, HE/IHC: Hematoxilin-eosin stain/Immunohistochemistry. ND: no data.

## Data Availability

The original contributions presented in the study are included in the article/Supplementary Material, further inquiries can be directed to the corresponding author/s.

## Supplementary Materials

The following supporting information can be downloaded at: https://www.mdpi.com/article/10.3390/vetsci12111101/s1, Table S1: Ranked generalized linear models (GLMs) with a binomial error distribution (logit link) assessing Toxoplasma gondii exposure (Serol) in chamois, based on different combinations of predictor variables. Models are ordered by the corrected Akaike’s Information Criterion (AICc), with corresponding log-likelihood (logLik), degrees of freedom (df), ΔAICc (delta), and Akaike weights (weight); Table S2: Model-averaged coefficients derived from generalized linear models (GLMs) with a binomial error distribution (logit link) assessing *Toxoplasma gondii* seroprevalence in chamois. Estimates are based on full model averaging across the candidate model set with corrected Akaike’s Information Criterion (AICc) values within ΔAICc < 2 of the top-ranked model. Reported values include parameter estimates (Estimate), unconditional standard errors (Std. Error), adjusted standard errors (Adjusted SE), z statistics, and associated p values. The code * indicates significant *p*-values.

## Abbreviations

The following abbreviations are used in this manuscript:

NGR	National Game Reserve
IHA	indirect hemagglutination
LA	latex agglutination
FA	direct immunofluorescence
MAT	Modified Agglutination Test
DAT	Direct Agglutination Test

## References

[1] Dubey J.P. (2021). Toxoplasmosis of Animals and Humans.

[2] Elmore S.A., Samelius G., Al-Adhami B., Huyvaert K.P., Bailey L.L., Alisauskas R.T., Gajadhar A.A., Jenkins E.J. (2016). Estimating *Toxoplasma gondii* exposure in arctic foxes (*Vulpes lagopus*) while navigating the imperfect world of wildlife serology. J. Wildl. Dis..

[3] Jensen S.K., Nymo I.H., Forcada J., Godfroid J., Hall A. (2012). Prevalence of *Toxoplasma gondii* antibodies in pinnipeds from Antarctica. Vet. Rec..

[4] Oksanen A., Asbakk K., Prestrud K.W., Aars J., Derocher A.E., Tryland M., Wiig O., Dubey J.P., Sonne C., Dietz R. (2009). Prevalence of antibodies against *Toxoplasma gondii* in polar bears (*Ursus maritimus*) from Svalbard and East Greenland. J. Parasitol..

[5] Prestrud K.W., Asbakk K., Fuglei E., Mørk T., Stien A., Ropstad E., Tryland M., Gabrielsen G.W., Lydersen C., Kovacs K.M. (2007). Serosurvey for *Toxoplasma gondii* in arctic foxes and possible sources of infection in the high Arctic of Svalbard. Vet. Parasitol..

[6] Corlatti L., Cotza A., Nelli L. (2021). Linking alternative reproductive tactics and habitat selection in Northern chamois. Ecol. Evol..

[7] Crampe J.P., Bon R., Gerard J.F., Serrano E., Caens P., Florence E., Gonzalez G. (2007). Site fidelity, migratory behaviour, and spatial organization of female isards (*Rupicapra pyrenaica*) in the Pyrenees National Park, France. Can. J. Zool..

[8] Ruiz-Olmo J., Such-Sanz A., Camps D., Sayol F., Batet T., Vilella M., Salvador S., Ruiz-Olmo J., Camps D. (2023). Grans Mamífers de Catalunya i Andorra: Distribució, Biologia, Ecologia i Convervació.

[9] Such-Sanz A., Ruiz-Olmo J., Sayol F., Batet T., Salvador S., Vilella M., Federico P., Campsolinas A., Piñol C., Ruiz-Olmo J., Camps D. (2023). Felis silvestris. Grans Mamífers de Catalunya i Andorra: Distribució, Biologia, Ecologia i Convervació.

[10] Castro-Scholten S., Cano-Terriza D., Jiménez-Ruiz S., Almería S., Risalde M.A., Vicente J., Acevedo P., Arnal M.C., Balseiro A., Gómez-Guillamón F. (2021). Seroepidemiology of *Toxoplasma gondii* in wild ruminants in Spain. Zoonoses Public Health.

[11] Gotteland C., Aubert D., Gibert P., Moinet M., Klein F.O., Game Y., Villena I., Gilot-Fromont E. (2014). Toxoplasmosis in natural populations of ungulates in France: Prevalence and spatiotemporal variations. Vector Borne Zoonotic Dis..

[12] Formenti N., Trogu T., Pedrotti L., Gaffuri A., Lanfranchi P., Ferrari N. (2015). *Toxoplasma gondii* infection in alpine red deer (*Cervus elaphus*): Its spread and effects on fertility. PLoS ONE..

[13] Formenti N., Gaffuri A., Trogu T., Viganò R., Ferrari N., Lanfranchi P. (2016). Spread and genotype of *Toxoplasma gondii* in naturally infected alpine chamois (*Rupicapra r. rupicapra*). Parasitol. Res..

[14] Crotta M., Pellicioli L., Gaffuri A., Trogu T., Formenti N., Tranquillo V., Luzzago C., Ferrari N., Lanfranchi P. (2022). Analysis of seroprevalence data on Hepatitis E virus and *Toxoplasma gondii* in wild ungulates for the assessment of human exposure to zoonotic meat-borne pathogens. Food. Microbiol..

[15] Marreros N., Hüssy D.H., Albini S., Frey C.F., Abril C., Vogt H.R., Holzwarth N., Wirz-Dittus S., Friess M., Engels M. (2011). Epizootiologic investigations of selected abortive agents in free-ranging alpine ibex (*Capra ibex ibex*) in Switzerland. J. Wildl. Dis..

[16] Gaffuri A., Giacometti M., Tranquillo V.M., Magnino S., Cordioli P., Lanfranchi P. (2006). Serosurvey of roe deer, chamois and domestic sheep in the central Italian Alps. J. Wildl. Dis..

[17] Ferroglio E., Bosio F., Trisciuoglio A., Zanet S. (2014). *Toxoplasma gondii* in sympatric wild herbivores and carnivores: Epidemiology of infection in the Western Alps. Parasites Vectors.

[18] Vengušt G., Kuhar U., Jerina K., Švara T., Gombač M., Bandelj P., Vengušt D.Ž. (2022). Passive Disease Surveillance of Alpine Chamois (*Rupicapra r. rupicapra*) in Slovenia between 2000 and 2020. Animals.

[19] National Air and Space Administration (NASA) Socioeconomic Data and Applications Center (SEDAC): Gridded Population of the World. https://ciesin.columbia.edu/content/data.

[20] Aegerter J., Fouracre D., Smith G.C. (2017). A First Estimate of the Structure and Density of the Populations of Pet Cats and Dogs across Great Britain. PLoS ONE.

[21] Simon J.A., Kurdzielewicz S., Jeanniot E., Dupuis E., Marnef F., Aubert D., Villena I., Poulle M.-L. (2017). Spatial Distribution of Soil Contaminated with *Toxoplasma Gondii* Oocysts in Relation to the Distribution and Use of Domestic Cat Defecation Sites on Dairy Farms. Int. J. Parasitol..

[22] Corlatti L., Gugiatti A., Imperio S. (2015). Horn growth patterns in Alpine chamois. Zoology.

[23] Kavčić K., Corlatti L., Safner T., Budak N., Šprem N. (2020). Contrasting patterns of sexually selected traits in Mediterranean and continental populations of European mouflon. Ecol. Evol..

[24] Sivachelvan M.N., Ghali Ali M., Chibuzo G.A. (1996). Foetal age estimation in sheep and goats. Small Rumin. Res..

[25] Dubey J.P., Desmonts G. (1987). Serological responses of equids fed *Toxoplasma gondii* oocysts. Equine Vet. J..

[26] Ajzenberg D., Lamaury I., Demar M., Vautrin C., Cabié A., Simon S., Nicolas M., Desbois-Nogard N., Boukhari R., Riahi H. (2016). Performance Testing of PCR Assay in Blood Samples for the Diagnosis of Toxoplasmic Encephalitis in AIDS Patients from the French Departments of America and Genetic Diversity of *Toxoplasma Gondii*: A Prospective and Multicentric Study. PLoS Negl. Trop. Dis..

[27] Galal L., Schares G., Stragier C., Vignoles P., Brouat C., Cuny T., Dubois C., Rohart T., Glodas C., Dardé M.L. (2019). Diversity of *Toxoplasma Gondii* Strains Shaped by Commensal Communities of Small Mammals. Int. J. Parasitol..

[28] Kosmidis I., Kenne Pagui E.C., Sartori N. (2020). Mean and median bias reduction in generalized linear models. Stat. Comput..

[29] Johnson J.B., Omland K.S. (2004). Model selection in ecology and evolution. Trends Ecol. Evol..

[30] R Core Team (2025). R: A Language and Environment for Statistical Computing.

[31] Stevenson M., Nunes T., Sanchez J., Thornton R., Reiczigel J., Robison-Cox J., Sebastiani P., Solymos P., Yoshida K., Firestone S. (2023). epiR: Tools for the Analysis of Epidemiological Data.

[32] Bartoń K. (2023). MuMIn: Multi-Model Inference.

[33] Kosmidis I. (2025). brglm2: Bias Reduction in Generalized Linear Models.

[34] Basso W., Holenweger F., Schares G., Müller N., Campero L.M., Ardüser F., Moore-Jones G., Frey C.F., Zanolari P. (2022). *Toxoplasma gondii* and *Neospora caninum* infections in sheep and goats in Switzerland: Seroprevalence and occurrence in aborted foetuses. Food Waterborne Parasitol..

[35] Marco I., Velarde R., López-Olvera J.R., Cabezón O., Pumarola M., Lavín S. (2009). Systemic toxoplasmosis and Gram-negative sepsis in a southern chamois (*Rupicapra pyrenaica*) from the Pyrenees in northeast Spain. J. Vet. Diagn. Invest..

[36] Dámek F., Swart A., Waap H., Jokelainen P., Le Roux D., Deksne G., Deng H., Schares G., Lundén A., Álvarez-García G. (2023). Systematic Review and Modelling of Age-Dependent Prevalence of *Toxoplasma gondii* in Livestock, Wildlife and Felids in Europe. Pathogens.

[37] Zhu S., Shapiro K., VanWormer E. (2022). Dynamics and epidemiology of *Toxoplasma gondii* oocyst shedding in domestic and wild felids. Transbound Emerg. Dis..

[38] Zhu S., VanWormer E., Shapiro K. (2023). More people, more cats, more parasites: Human Population Density and Temperature Variation Predict Prevalence of *Toxoplasma gondii* Oocyst Shedding in Free-ranging Domestic and Wild Felids. PLoS ONE.

[39] Shapiro K., Bahia-Oliveira L., Dixon B., Dumètre A., deWit L.A., VanWormer E., Villena I. (2019). Environmental transmission of *Toxoplasma gondii*: Oocysts in water, soil and food. Food Waterborne Parasitol..

[40] Matas Méndez P., Fuentes Corripio I., Montoya Matute A., Bailo Barroso B., Grande Gómez R., Apruzzese Rubio A., Ponce Gordo F., Mateo Barrientos M. (2023). Prevalence of *Toxoplasma gondii* in endangered wild felines (*Felis silvestris* and *Lynx pardinus*) in Spain. Animals.

[41] Sobrino R., Cabezón O., Millán J., Pabón M., Arnal M.C., Luco D.F., Gortázar C., Dubey J.P., Almeria S. (2007). Seroprevalence of *Toxoplasma gondii* antibodies in wild carnivores from Spain. Vet. Parasitol..

[42] Candela M.G., Pardavila X., Ortega N., Lamosa A., Mangas J.G., Martínez-Carrasco C. (2019). Canine distemper virus may affect European wild cat populations in Central Spain. Mamm. Biol..

[43] Miró G., Rupérez C., Checa R., Gálvez R., Hernández L., García M., Canorea I., Marino V., Montoya A. (2014). Current status of *L. infantum* infection in stray cats in the Madrid region (Spain): Implications for the recent outbreak of human leishmaniosis?. Parasites Vectors.

[44] Montoya A., García M., Gálvez R., Checa R., Marino V., Sarquis J., Barrera J.P., Rupérez C., Caballero L., Chicharro C. (2018). Implications of zoonotic and vector-borne parasites to free-roaming cats in central Spain. Vet. Parasitol..

[45] Marbella D., Santana-Hernández K.M., Rodríguez-Ponce E. (2022). Small islands as potential model ecosystems for parasitology: Climatic influence on parasites of feral cats. J. Helminthol..

[46] Millán J., Candela M.G., Palomares F., Cubero M.J., Rodríguez A., Barral M., de la Fuente J., Almería S., León-Vizcaíno L. (2009). Disease threats to the endangered Iberian lynx (*Lynx pardinus*). Vet. J..

[47] Hatam-Nahavandi K., Calero-Bernal R., Rahimi M.T., Pagheh A.S., Zarean M., Dezhkam A., Ahmadpour E. (2021). *Toxoplasma gondii* infection in domestic and wild felids as public health concerns: A systematic review and meta-analysis. Sci. Rep..

[48] Garcia-Sanchez P., Romero-Trancón D., Falces-Romero I., Navarro Carrera P., Ruiz-Carrascoso G., Carmena D., Casares Jiménez M., Rivero-Juárez A., Moya L., Rodón J. (2024). Zoonosis screening in Spanish immunocompromised children and their pets. Front. Vet. Sci..

[49] Peris M.P., Planas S., Langa J., Laborda A., Castillo J.A., Gracia M.J. (2024). Seroprevalence of zoonotic pathogens in stray cats in an urban area of northeast Spain. Vet. Parasitol. Reg. Stud. Rep..

[50] Villanueva-Saz S., Martínez M., Giner J., González A., Tobajas A.P., Pérez M.D., Lira-Navarrete E., González-Ramírez A.M., Macías-León J., Verde M. (2023). A cross-sectional serosurvey of SARS-CoV-2 and co-infections in stray cats from the second wave to the sixth wave of COVID-19 outbreaks in Spain. Vet. Res. Commun..

[51] Candela M.G., Fanelli A., Carvalho J., Serrano E., Domenech G., Alonso F., Martínez-Carrasco C. (2022). Urban landscape and infection risk in free-roaming cats. Zoonoses Public Health.

[52] Villanueva-Saz S., Giner J., Tobajas A.P., Pérez M.D., González-Ramírez A.M., Macías-León J., González A., Verde M., Yzuel A., Hurtado-Guerrero R. (2022). Serological evidence of SARS-CoV-2 and co-infections in stray cats in Spain. Transbound Emerg. Dis..

[53] García-Bocanegra I., Dubey J.P., Martínez F., Vargas A., Cabezón O., Zorrilla I., Arenas A., Almería S. (2010). Factors affecting seroprevalence of *Toxoplasma gondii* in the endangered Iberian lynx (*Lynx pardinus*). Vet. Parasitol..

[54] Millán J., Cabezón O., Pabón M., Dubey J.P., Almería S. (2009). Seroprevalence of *Toxoplasma gondii* and *Neospora caninum* in feral cats (*Felis silvestris catus*) in Majorca, Balearic Islands, Spain. Vet. Parasitol..

[55] Roelke M.E., Johnson W.E., Millán J., Palomares F., Revilla E., Rodríguez A., Calzada J., Ferreras P., León-Vizcaíno L., Delibes M. (2008). Exposure to disease agents in the endangered Iberian lynx (*Lynx pardinus*). Eur. J. Wildl. Res..

[56] Miró G., Montoya A., Jiménez S., Frisuelos C., Mateo M., Fuentes I. (2004). Prevalence of antibodies to *Toxoplasma gondii* and intestinal parasites in stray, farm and household cats in Spain. Vet. Parasitol..

[57] Gauss C.B., Almería S., Ortuño A., Garcia F., Dubey J.P. (2003). Seroprevalence of *Toxoplasma gondii* antibodies in domestic cats from Barcelona, Spain. J. Parasitol..

[58] Aparicio Garrido J., Cour Boveda L., Berzosa Aguilar M., Pareja Miralles J. (1972). Study on the epidemiology of toxoplasmosis. Infection in the domestic cat in suburbs of Madrid. Serological and copro-parasitological study. Med. Trop..

[59] Elmore S.A., Jenkins E.J., Huyvaert K.P., Polley L., Root J.J., Moore C.G. (2012). *Toxoplasma gondii* in circumpolar people and wildlife. Vector Borne Zoonotic Dis..

