# Supplementary material for: Exposure of Wild Ruminants to Toxoplasma gondii in Alpine Ecosystems, NE Spain
Source: Vet Sci. 2025 Nov 18;12(11):1101. doi: 10.3390/vetsci12111101 (PMC12656981; doi:10.3390/vetsci12111101)
Supplement: Supplementary file 1 [file vetsci-12-01101-s001.zip › vetsci-3921880-supplementary.pdf]

Table S1. Ranked generalized linear models (GLMs) with a binomial error distribution (logit link) assessing *Toxoplasma gondii* exposure (Serol) in chamois, based on different combinations of predictor variables. Models are ordered by the corrected Akaike's Information Criterion (AICc), with corresponding log-likelihood (logLik), degrees of freedom (df), ΔAICc (delta), and Akaike weights (weight).

Model	df	logLik	AICc	delta	weight
Serol ~ Method + Zone	6	-188.27	388.64	0	0.39
Serol ~ Method + Zone + Age	7	-187.8	389.71	1.08	0.23
Serol ~ Method + Zone + Sex	7	-187.91	389.93	1.3	0.2
Serol ~ Method + Zone + Age + Sex	8	-187.47	391.08	2.44	0.11
Serol ~ Method	3	-193.9	393.83	5.19	0.03
Serol ~ Method + Age	4	-193.1	394.23	5.6	0.02
Serol ~ Method + Sex	4	-193.92	395.87	7.24	0.01
Serol ~ Method + Age + Sex	5	-193.11	396.29	7.65	0.01

Table S2. Model-averaged coefficients derived from generalized linear models (GLMs) with a binomial error distribution (logit link) assessing Toxoplasma gondii seroprevalence in chamois. Estimates are based on full model averaging across the candidate model set with corrected Akaike's Information Criterion (AICc) values within ΔAICc < 2 of the top-ranked model. Reported values include parameter estimates (Estimate), unconditional standard errors (Std. Error), adjusted standard errors (Adjusted SE), z statistics, and associated p values. The code * indicates significant p-values.

Term	Estimate	Std. Error	Adjusted SE	z statistic	p value
(Intercept)	-2.147	0.541	0.542	3.961	<0.001 *
Zone RNC Cadí	-0.42	0.454	0.455	0.924	0.356
Zone RNC Cerdanya-Alt Urgell	0.005	0.572	0.573	0.008	0.993
Zone RNC Freser-Setcases	-1.204	0.434	0.434	2.772	0.006 *
Method IDEXX	-2.952	1.444	1.446	2.042	0.041 *
Method MAT	0.333	0.395	0.395	0.844	0.399
Age (2-year index)	0.021	0.049	0.049	0.417	0.677
Sex Male	-0.064	0.184	0.184	0.346	0.729
